# Social inclusion of students with special educational needs assessed by the Inclusion of Other in the Self scale

**DOI:** 10.1371/journal.pone.0250070

**Published:** 2021-04-28

**Authors:** Jana Vyrastekova

**Affiliations:** Institute for Management Research, Nijmegen Center for Economics, Radboud University, Nijmegen, The Netherlands; University of South Australia, AUSTRALIA

## Abstract

How does the participation of students with special educational needs (SEN) in mainstream education affect their social inclusion? We introduce a single-item pictorial measure, the Inclusion of Other in the Self (IOS), to compare the social inclusion of SEN students attending mainstream regular schools to social inclusion of SEN students attending special schools. We collected responses from 138 parents of SEN students aged 4–20, to obtain data on the loneliness, friendships and social inclusion of SEN students. The parents of SEN students attending regular schools did not perceive their children to be less included than parents of SEN students attending special schools. School context decreased SEN students’ perceived loneliness independent of the school type. And while most SEN students’ friendships were formed at school, SEN students attending regular schools had more friends, and these were more likely to live in the same neighborhood. Overall, the social inclusion of SEN students across school types was not affected by the school type, only by the school’s inclusive characteristics.

## Introduction

The participation of students with special educational needs (SEN) in regular schools is frequently disputed on the grounds that their social inclusion is failing. SEN students in regular schools were identified as being more lonely than their non-SEN classmates [1–3], having fewer friends and interactions with peers [4–6], and being more likely to be bullied [7]. These concerns arise from studies comparing SEN students to non-SEN students, using measures of social inclusion that are based on sociometric methods and observational data. Social inclusion is defined in these studies as the presence of reciprocal friendships, interactions between SEN and non-SEN students, the social status of SEN students as perceived by non-SEN students, and the acceptance of SEN students by their classmates [8].

We propose that comparing the social inclusion of SEN students across educational contexts is a relevant but missing piece of evidence in the current discussion. To evaluate the impact of including SEN students in regular schools as an alternative to their schooling in segregated context of special schools, we have to compare their social inclusion across the school contexts, rather than to compare the social inclusion of SEN students to that of non-SEN students. We offer such a comparison across school contexts, and introduce a new approach to assessing social inclusion based on a subjective perspective of social inclusion. Indeed, recent research shows that although SEN and non-SEN students achieve different outcomes on sociometric measures in regular schools, their perceptions of quality of friendships do not differ [9]. This suggests that the currently used sociometric methods for assessing social inclusion might underestimate the sense of belonging and social inclusion that SEN students experience in regular schools. Therefore, we also offer a new measure of social inclusion, and respond to the call to create new methods of evaluation of social inclusion [8].

Our approach is inspired by the literature on subjective well-being [10–12], which has contributed significantly to our understanding how the ultimate goal of economic progress—human well-being–is linked to its traditionally frequently used economic indicators, like income. If the ultimate goal is to achieve the highest possible well-being for an individual, why not assess this well-being directly? Using this approach, it has become apparent that an increase in income does not necessarily translate into an increase in all aspects of well-being [13]. Furthermore, by directly measuring well-being, the researchers have been able to study factors that underlie well-being (e.g., social relationships) and the impact well-being has on individual’s health or labor market outcomes. The challenge of working with subjective measures of social inclusion is comparable to the challenge that the researchers have faced when introducing subjective well-being measures in economics [14]. However, there are likely benefits from accepting such a challenge. The new insights that we have gained thanks to the measures of subjective well-being put forth in the literature have resulted in contributions to the formulation of economic policy goals [15] and have motivated policy-makers to consider subjective well-being complementary to the income measures of economic success.

What is more, the foundation for applying a subjective perspective on all aspects of well-being of SEN students, including social inclusion, is found in the Article 7.3 of the United Nations Convention on the Rights of Persons with Disabilities [16] stating that: *“States Parties shall ensure that children with disabilities have the right to express their views freely on all matters affecting them*, *their views being given due weight in accordance with their age and maturity*, *on an equal basis with other children*, *and to be provided with disability and age-appropriate assistance to realize that right*.*”* We therefore propose to further the understanding of factors promoting social inclusion in education by adopting a well-validated, simple and easy to administer pictorial measure Inclusion of Other in the Self Scale (IOS) [17] as a subjective measure of social inclusion. In this study, we use this measure to address how including of SEN students in regular schools affects their social inclusion compared to SEN students in segregated special schools.

This research contributes to the vibrant ongoing normative and empirical discussions about the impact of inclusion in education [18–20]. Inclusion means more than integration of the SEN students in the mainstream system, by placing SEN students in the mainstream schools without a transformation of the education system. The UN Convention on the Human Rights of Persons with Disabilities, Article 24 [21] recognizes the right of inclusive education for all learners, calling for an education system adapted to the needs of all learners. This legal foundation of inclusion in education has driven efforts to include SEN students in schools with all learners worldwide. However, the success of social inclusion of SEN students, with its goal: "*to facilitate true social inclusion a person needs to be both connected and have a sense of belonging*” [22] remains disputed, although there is a range of positive findings in support of inclusive education.

Early inclusion in education has, for example, been found to increase mutual understanding [23, 24]. In addition, the academic achievements of SEN students are higher in regular schools than in special segregated schools [25–27] and the academic achievements of non-SEN students are not negatively affected by inclusion; on the contrary, non-SEN students perform slightly better in inclusive settings with SEN classmates [28–30]. There is also a measurable positive impact on the number of reciprocal friendships and peer acceptance [31] for non-SEN students attending inclusive classrooms. Despite this evidence on the positive impact of inclusion in education, however, it remains unclear whether including SEN students in regular schools promotes or harms their social inclusion.

Our study is based in the Netherlands which ratified the United Nations Convention on the Rights of People with Disabilities in 2016. Although formally, this awards the right of inclusive education to all learners, the education of SEN students in the Netherlands at the time of our study still took place in two parallel systems: in special schools, with access restricted by eligibility (i.e. by proof of special educational needs), and in regular schools, which occasionally integrate SEN students. We use the existence of this dual system of education for SEN students in the Netherlands, to assess whether their social inclusion varies with the school type.

The contribution of our study is twofold. First, we propose a simple pictorial measure of relationship with others, the IOS Scale [17], to assess the social inclusion of SEN students. This novel approach to social inclusion focuses on the perception of being included, rather than on demonstrations of inclusion and evaluations via interactions with others. Second, we use this measure of social inclusion to address our research question. How does the social inclusion of SEN students differ across school types, when comparing SEN students attending regular schools to SEN students attending special schools? Data on friendships arising in school and outside of school, together with a short measure of loneliness at home and at school further clarify the impact of including SEN students in regular schools on their social inclusion. This approach offers a new perspective on the impact of including SEN students in the regular schools.

## Theory

Inclusive education stands high on the international education policy agenda [32]. Article 24 of the United Nations Convention on the Rights of Persons with Disabilities [16] states that the States Parties must guarantee that: “*[p]ersons with disabilities can access an inclusive*, *quality and free primary education and secondary education on an equal basis with others in the communities in which they live*.” Inclusive education is further described in the Convention as a way to achieve the social inclusion of persons with disabilities [33].

The country where we performed our study, the Netherlands, ratified the United Nations Convention on the Rights of Persons with Disabilities in 2016 but has maintained a dual system of special and mainstream regular schools [34]. The primary phase in the Dutch education system comprises children 4–12 years of age, and is followed by obligatory secondary education [35]. Since 2014, when the Appropriate Education Act (Wet Passend Onderwijs) was introduced in the Netherlands, local school consortia have been made responsible for offering an adequate education to every student with the hope of promoting inclusive education. Contrary to the expectations, the inclusion of SEN students in regular schools due to this act has not generally increased [34], although there were significant regional variations associated with varying models of financing the support of SEN students [36]. In 2016/17, about 2% of primary school students in the Netherlands attended special schools [37]. When the special educational needs of a student are established by a committee of the regional school consortium, a SEN student can be placed either at a special school or, conditional upon approval by the school, at a regular school, in which case regionally varying financing models are used to finance the placement. This coexistence of regular and special school placement of SEN students in the Dutch system allowed us to address the impact of the alternative student placement on their social inclusion. Since the placement is not random, and may depend on the school and student characteristics, we account for both these factors in our analysis.

Social inclusion is a complex concept, broadly understood as an interaction between interpersonal relationships and community participation [38]. Assessing the achievement of such a broad concept is difficult, and it is therefore useful to operationalize social inclusion via its relational aspects, namely the feeling of connectedness and sense of belonging [39]. Feeling of connectedness and sense of belonging represent basic human motivations [40] and address the extent to which a person identifies as a part of his or her own social context and feels as belonging to it, instead of feeling alone. Feelings of connectedness and a sense of belonging therefore stand central in perceiving social inclusion.

Social inclusion is thus not only about being a part of something as a passive participant or bystander but also about perceiving oneself as connected and emotionally positively affected. To give an example of the difference between social inclusion and participation, a person may be a member of a hobby group or a class at school, perceiving oneself as part of a group, but experience the lack of connection on the emotional level, and feel lonely. Mere participation is not sufficient for being socially included. Higher levels of belonging, and perceived social inclusion, are associated with lower levels of loneliness.

Another aspect that is considered a demonstration of social inclusion is friendships, often defined as reciprocal relationships within a dyad or a group. Friendships fulfill multiple functions in life: they provide support, access to information, safety, entertainment, and health [41, 42]. It has been proposed that children and young adults with disabilities are particularly dependent on friendships arising at school due to the restrictions they might experience in other types of social contacts, such as after-school activities or in joining sports or hobby clubs [43, 44]. Social scientists propose that homophily, associating with others similar to oneself, serves as a strong organizing principle in social relations and forming friendships. In short: “*Similarity breeds connection*” [45]. SEN students might experience low homophily in regular schools, among a majority of non-SEN students. Additionally, social comparisons could negatively affect SEN students’ self-concept in regular schools, due to exposure to peers with social and cognitive skills unaffected by the SEN student’s characteristics [46].

On the other hand, it is also possible that the selected subgroup of SEN students that is accepted by the regular schools in a dual educational system coincides with the group of the most socially adjusted SEN students. If this selection effect is present, we would expect SEN students attending regular schools to feel less lonely, and be more included than SEN students attending special schools, however not only at school, but also at home. The measure of loneliness at home is a control variable for the selection effect possibly accompanying the admission of SEN students to regular schools due to the characteristics of the SEN students associated with social inclusion.

Based on this exposition, we can identify multiple mechanisms by which including SEN students in regular schools could result in a harsher social environment for them, negatively affect their ability to form friendships, drive feelings of loneliness at school, and result in low social inclusion, when controlling for the possible selection effect via individual student and school characteristic. We test the hypotheses that SEN students attending regular schools achieve lower social inclusion, have fewer friends and feel more lonely at school, compared to SEN students attending special schools, using IOS Scale as a measure of social inclusion.

## Method

### Participants

The data collection took place between December 2016 and March 2017 and was organized via an online questionnaire disseminated by three Dutch organizations representing the interests of citizens with disabilities or chronic disease, or their parents: IederIn (Dutch umbrella organization for people with a physical disability, mental disability or chronic illness), Dutch Patient Association (https://www.patientenfederatie.nl/), and National Platform of Mental Health (http://www.platformggz.nl/lpggz/). This helped to guarantee the credibility of the data collection. The questionnaire was posted on the social media pages of these organizations, meaning that the sample cannot be considered representative of the whole population. This study participants represent a group of parents of SEN students (aged 4–20) seeking information provided by these organizations.

Prior to the data collection, we obtained approval for this study from the Ethical Committee of the Faculty of Management, Radboud University, The Netherlands. Each participant, the parent of a SEN student, gave informed consent for the data provided to be used for this research, by clicking on the approval box before starting the questionnaire. In total, 138 parents of SEN students (aged 4–20) who completed the questionnaire gave informed consent to participate and have their data used for this research. Among them, 68 respondents reported about a SEN student attending a special school, and 70 about a SEN student attending a regular school.

Table 1 contains the characteristics of the SEN students by school type, reported upon by their parents. These SEN students had various types of disability, and the mode was one type of disability. Both genders were represented, though the proportion of boys was somewhat higher (62%). A Mann-Whitney test indicated that age of SEN students did not significantly differ for regular schools (Mdn = 11) and for special schools (Mdn = 12), U(N_regular_ = 70, N_special_ = 68) = 2020.5, p = 0.124.

**Table 1:** Characteristics of the SEN students by school type.

	Total	Regular school	Special school
Number of respondents	138	70	68
Gender			
Girls	52	34	18
Boys	86	36	50
Age in years (range)	4–20	4–20	4–20
Age (average +/- st.dev)	11,6 +/- 3,9	11,2 +/- 3,9	12,1 +/- 3,9
Type of disability			
Visual	5	1	4
Hearing	10	4	6
Physical	28	13	15
Cognitive	29	4	25
Mental health conditions	49	25	24
Chronic disease	21	13	8
Other	50	26	24
Number of handicaps			
exactly 1	105	56	49
2 or more	33	14	19

### Measures

#### Inclusion of Other in the Self

**Fig 1:**
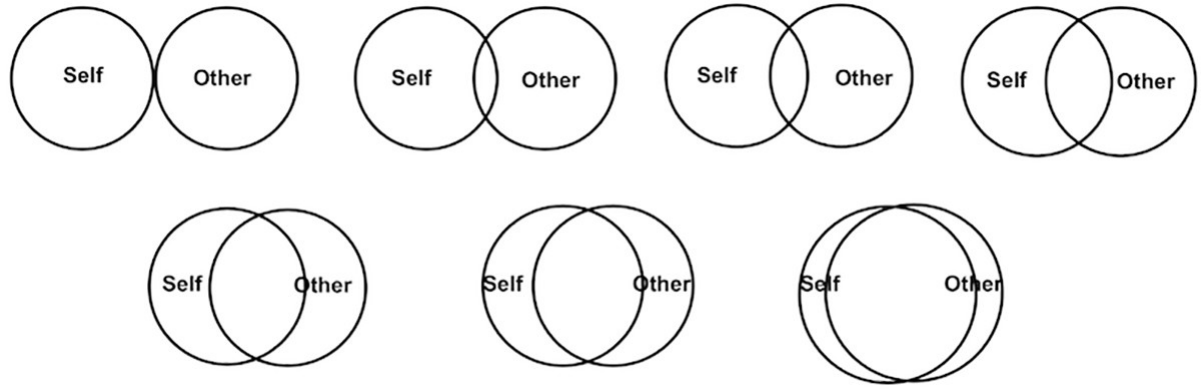
The Inclusion of Other in the Self scale based on Aron et al. (1992). In our study, “Self” referred to “My child” and “Other” referred to “Other students at school”.

We use the pictorial IOS Scale [17] as a measure of social inclusion. The single-item IOS pictorial scale consists of seven pictures, each presenting two circles. The two circles show increasing overlap starting from the first to the seventh picture (Fig 1). The extent of the overlap is intuitively understood by the respondents as the closeness of the relationship between the subjects presented in the two circles, for example between the responder and the “Other” identified in the circle; a higher overlap stands for a closer relationship. The respondent is asked to indicate which of the seven pictures best represents the relationship with the “Other”. In our study, the relationship between the SEN student and “Other” where this stands for “Other students at school” is reported by the parents of the SEN students. The IOS measure is understood in various contexts as a question about the closeness of relationship with others and being a part of the community; it is intuitive and performs reliably across contexts. Due to its simplicity, it has been proposed as a toolkit for younger audiences [47] and successfully applied to explain a broad range of relational behaviors such as citizenship [48], socially responsible decisions [49], or being a part of a community [50].

The IOS measure has recently attracted the attention of behavioral scientists studying the impact of social relationships. Gächter et al. [51] constructed an Index of Relationship Closeness based on several social relationships measuring tools and showed that IOS is highly correlated with this index. They concluded that IOS is a “psychologically meaningful and highly reliable measure of subjective closeness of relationships”, and effectively replaces more extensive and complex measures. Based on this research validating IOS as a measure of a sense of belonging and connectedness with others, we propose that the IOS measure is suitable to capture these elements of social inclusion.

Our study obtained data on the social inclusion of SEN students using the IOS measure from parental proxy reports. We relied on the existing evidence suggesting that although parents may systematically under- or overestimate certain areas of child’s well-being, they also have a fairly good understanding of the child’s overall well-being and his/her relationship with peers [52]. To account for the parents’ imperfect insight into the social inclusion of their children, we systematically added an alternative answer “I do not know/I do not wish to answer this question” in the questionnaires when collecting the IOS Scale and likewise did so for the Loneliness measure discussed below.

#### Loneliness

Loneliness was assessed by a short 3-item Loneliness in children scale [53] composed of the answers to the questions: (1) I feel alone at school/at home; (2) I feel left out of things at school/at home; (3) I’m lonely at school/at home, measured with a 5-point Likert scale, and evaluated both for the school and home context, respectively. This very short scale is based on the original 24-item scale, has correlated with r = .84 with the full scale [54], was proposed as a promising measure of loneliness, and has been shown to link with self-reported loneliness [55]. We constructed the loneliness variable as the sum of the responses to the three questions quoted above. Loneliness has been linked to the perception of being rejected by others, and to social dissatisfaction [56]. By asking the loneliness question in two contexts, at school and at home, we could address how the school type affects loneliness, compared to the baseline loneliness experienced at home.

#### Friendships

We collected information on friendships arising in and outside of school by asking how many friends the SEN student had in each context. The friendships were reported by parents, without the researcher giving an explicit definition of what was understood as friendship. We also asked for the number of online friends, not to pollute the reports with friendships that might arise in a virtual context, but not lead to contact. While virtual friendships may represent an important source of social interaction, especially for disabled youth [57], we focused on friendships that open the possibility of social inclusion in the same neighborhood where the student lives. To this end, we also asked where most of the friends lived: in particular, we asked whether they lived in the same neighborhood at the SEN student, to address whether friendships started at school promote the creation of social networks in own community, in the place where the SEN students live.

#### School characteristics

The SEN students in our sample attended either a special or a regular school, and we categorized these schools by their formal status, as reported by the parents. Each school is thus either categorized as a special or a regular school. Special schools are schools that only admit SEN students, based on evidence of special educational needs. However, the schools can also differ in school culture, which may have impacted on the social inclusion of the SEN students. Therefore, we adopted twelve questions from the Index for Inclusion [58] to measure how inclusive the school is in its acceptance of diversity, its didactic choices, and the social environment it created. These selected questions (see the Supplementary files) capture how welcoming the school was, how it protected each child’s well-being, and made didactic choices for diversity and collaboration. The answers were obtained on a 5-point Likert scale, and summarized into a single variable, by summing up the responses in the variable SchoolInclusion, ranging from 12 to 60. The higher the value of the variable, the more inclusive the school was for all learners.

We also account for the possibility that parental reports might distort the actual inclusion as perceived by their children and this effect could be mediated by the parent’s perception of the school. For example, parents might be inflating the reports for students attending a regular school. This could happen if parents feel more accepted by their child’s school in the case of a regular school willing to accept a SEN student. The variable ParentalInclusion was based on answers to the question “The school works in partnership with me as parent/care-taker”on a 5-point Likert scale between Totally Disagree and Totally Agree, and was adopted to control for the possibility that parents’ reports on the degree of child inclusion were conflated by their own perceptions of how inclusive the school was.

#### Data analyses

This paper studies how SEN students’ social inclusion is affected by their school placement, in special or regular schools, along with loneliness and friendships arising at school and outside school. We first summarized each of the variables, and addressed whether SEN students in special and regulars schools differed in their subjectively perceived social inclusion, loneliness and friendships. We used Mann-Whitney U tests to detect the median differences in the distributions of social inclusion and loneliness under the two school types. The Spearman rank-based correlation coefficient was applied to correlate loneliness at home and school with the subjective perception of inclusion at school, and we tabulated the distribution on the friendships arising at school and outside school.

Our next step was to perform a regression analysis to address whether subjectively perceived social inclusion at school could be explained by the type of school, while controlling for loneliness at home, and friendships at school. We estimated an ordered logit model with the dependent variable being the IOS Scale, ranging from 1 (least included) to 7 (most included), including school type as the main variable. We accounted for the objective classification of schools by their admission criteria as regular of special schools, and also for the fact that these schools might differ in their school culture, as captured by the SchoolInclusion variable.

We also included the ParentalInclusion variable, which captured how included the parents themselves felt at the school of their own choice. In addition, individual student characteristics age, gender, and number of handicaps could affect inclusion, and were added in the regressions as variables Age, Female, and MoreThan1Handicap, respectively. Another bias would be if parents reported the child’s personality type: a socially well-adjusted student with many friends at home might be projected as such at school, even when this was incorrect. We controlled for this by the FriendsNotAtSchool variable, indicating the number of friends that the student had outside the school.

## Results

### Inclusion of Other in the Self scale

**Fig 2:**
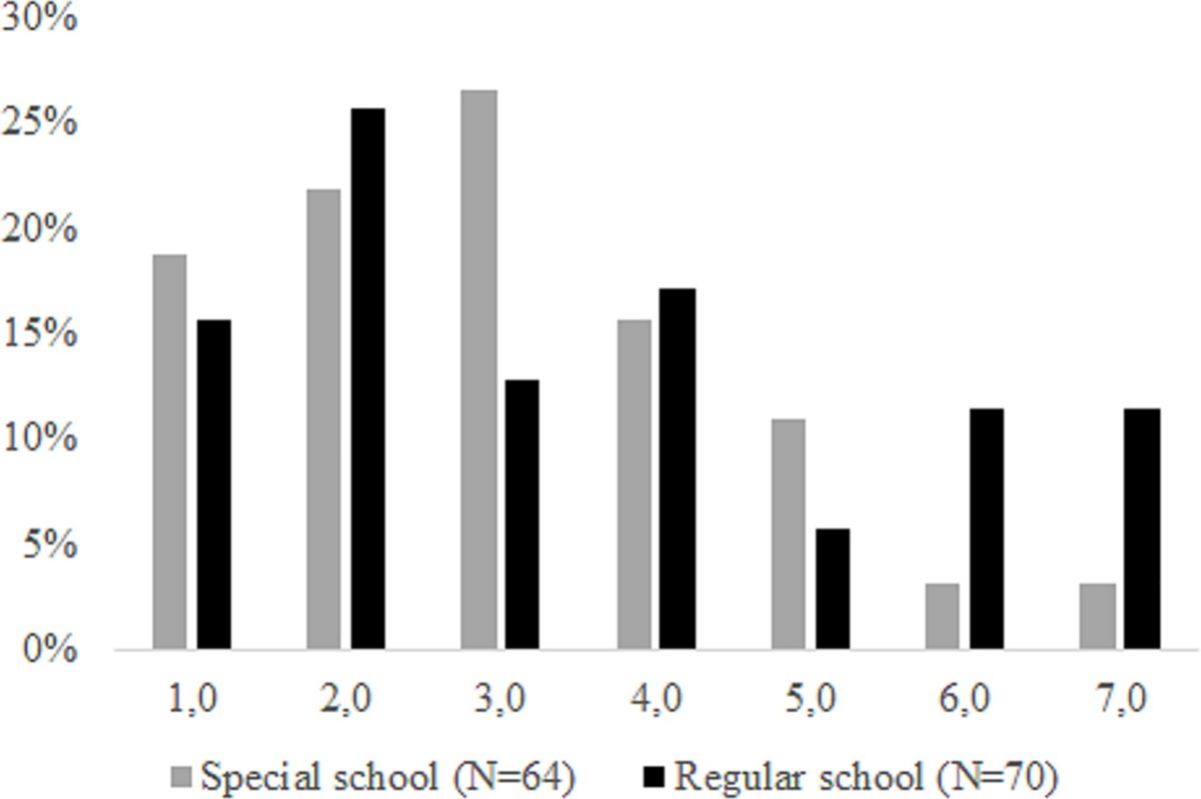
Inclusion of Other in the Self scale.

Fig 2 contains information on the main variable we introduced to measure social inclusion—the IOS Scale. Only 4/138 respondents indicated that the IOS question was too difficult to answer. Most of the responses referred to one of the three least-overlapping circles (responses 1, 2 or 3 in Fig 1), with 63% of SEN students from special schools and 46% of SEN students from regular schools associated with these three lowest levels of inclusion. Importantly, we found no significant difference in the reports for the SEN students attending regular and special schools. A Mann-Whitney test indicated that IOS did not significantly differ for SEN students attending regular schools (Mdn = 3) and for those attending special schools (Mdn = 3), U(N_regular_ = 70, N_special_ = 64) = 1967.5, p = 0.217. Reports on the IOS Scale did not support that including SEN students in regular schools was associated with them being perceived as less included by their parents, compared to the perceptions of parents of their special school attending peers.

### Loneliness

First, we studied the measure of loneliness of SEN students at home. We asked whether there were systematic differences between SEN students attending regular and special schools, to exclude a selection effect. A Mann-Whitney test indicated that SEN students attending regular schools did not significantly differ in how lonely their parents perceived them to be at home (Mdn = 12) from SEN students attending special schools (Mdn = 12), U (N_regular_ = 67, N_special_ = 66) = 2185.0, p = 0.679. Both groups were on average perceived to be on the lonely side, with an average short scale value of 9.6 vs. 10.6 for students of regular vs. special schools, respectively, where 9 was the neutral score. A Mann-Whitney test further showed that SEN students attending regular schools did not significantly differ in how lonely their parents perceived them to be at school (Mdn = 10) from SEN students attending special schools (Mdn = 12), U(N_regular_ = 66, N_special_ = 64) = 1708.0, p = 0.058.

Overall, parents of SEN students mostly perceived SEN students to be less lonely at school than at home, independent of school type, see Table 2 for distribution of the cases when a SEN student’s loneliness at school and at home was compared on an individual level, and classified as more, equally, of less lonely at school than at home, for both school types.

**Table 2:** Loneliness at school and at home.

	Special school	Regular school
More lonely at school than at home	22%	12%
Equally lonely	25%	20%
Less lonely at school than at home	53%	69%
N (respondents)	64	65

Furthermore, we addressed the link between the IOS measure and loneliness. As expected, we found that the IOS measure was highly and significantly correlated with a short measure of loneliness assessed in the same context (school), lending support to its validity (Spearman correlation coefficient 0.508, p = 0.000), while the association with loneliness in an unrelated context (at home) was much weaker (Spearman correlation coefficient 0.173, p = 0.048).

### Friendships

**Fig 3:**
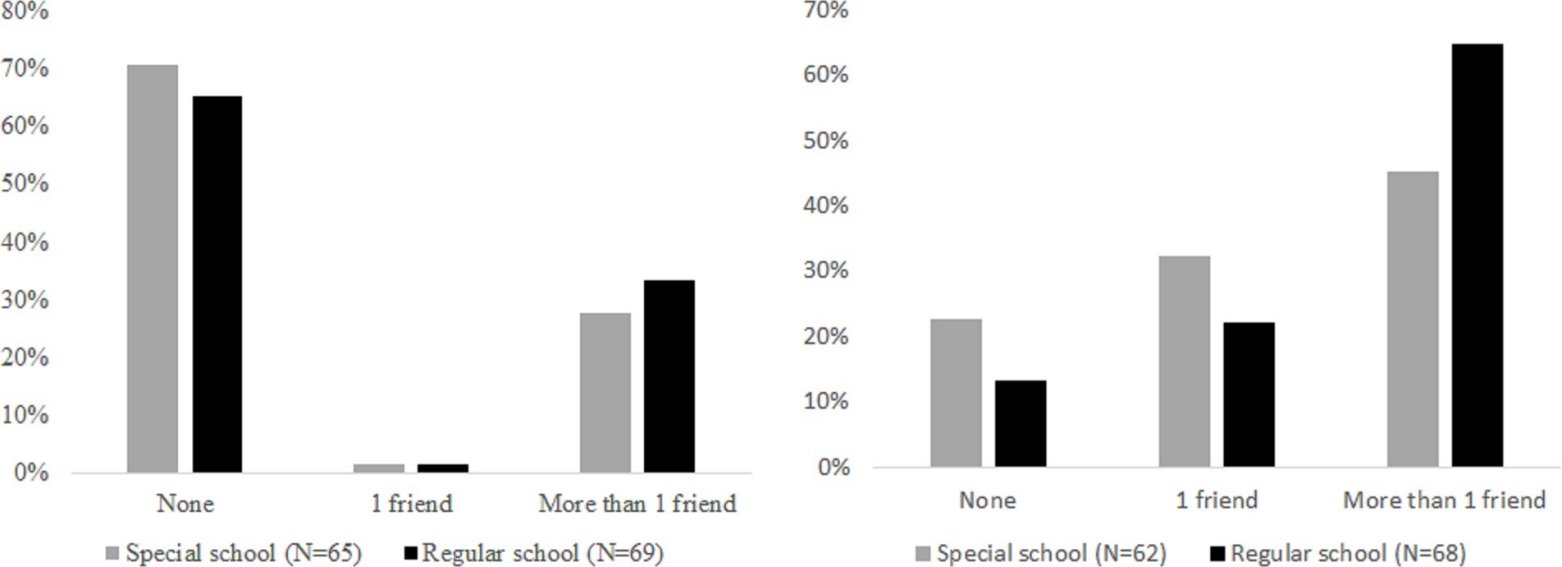
a. Friends not at school, by school type. b. Friends at school, by school type.

The information on the number of friends at school and outside school is summarized in Fig 3a and 3b, respectively, by presenting the number of friendships in three categories: no friend, one friend, and more than one friend. This approach is less sensitive to outliers than looking at the actual number of friends. Using an outlier-insensitive test on the number of friends, Mann-Whitney test indicated that SEN students at regular schools had significantly more friends at school (Mdn = 2) than SEN students at special schools (Mdn = 1), U(N_regular_ = 64, N_special_ = 61) = 1549.5, p = 0.043. But, Mann-Whitney test also indicated that there was no difference in the number of friends arising outside school for SEN students at regular schools (Mdn = 0) and for SEN students at special schools (Mdn = 0), U(N_regular_ = 69, N_special_ = 65) = 2066.5, p = 0.344. We observed that most of the friendships of SEN students were formed at school, independent of the school type, but more friendship were formed at regular schools than at special schools.

We further observed that friends of SEN students attending regular schools were significantly more likely to live in the same neighborhood, in 45.7% (32/70) of cases, than friends of SEN students attending special schools, in 14.7% (10/68) of cases (Fisher exact test, p = 0.000). This observation is easily understood in the local context of our study, the Netherlands, where special schools have a larger catchment area than regular schools. Consequently, if friendships mostly arise at school, friends of SEN students attending regular schools will be more likely coming from the same neighborhood than friends of SEN students attending special schools.

### School characteristics

A Mann-Whitney test indicated that regular schools in our sample scored significantly higher on the variable SchoolInclusion (Mdn = 41) than special schools in our sample (Mdn = 45), U(N_regular_ = 55, N_special_ = 48) = 965.5, p = 0.019.

### Regression analysis

We have observed that school type did not affect how socially included the SEN students were as perceived by their parents, but we have only looked at bivariate analysis so far. Table 3 shows the multinomial logit regression results, supporting our observation that IOS measure, the dependent variable, was not negatively affected by the attendance of a regular school. Indeed, the school type seemed not to be related to the SEN students’ social inclusion, as reported by their parents. Neither the school type, nor the student’s individual characteristics affected the level of IOS reported. The results also suggested that parents did not systematically perceive social inclusion through the lens of being more accepted as a partner at school. The variable ParentalInclusion was not significant. The only variable that affected the IOS measure was the SchoolInclusion variable, addressing the school’s inclusive culture. Including an interaction between the school type and SchoolInclusion variable in the regressions remained insignificant. We therefore omitted this interaction from the reported regression models.

**Table 3:** Ordered logit regression explaining Inclusion of Other in the Self.

	(a)	(b)	(c)	(d)	(e)
Regular (0 = No, 1 = Yes)	0.173		0.581	0.574	0.498
	(0.300)		(0.360)	(0.360)	(0.407)
SchoolInclusion		0.042	0.049	0.080	0.086
		(0.016)**	(0.018)**	(0.032)**	(0.035)**
ParentalInclusion				-0.315	-0.289
				(0.263)	(0.273)
Female (0 = No, 1 = Yes)					0.619
					(0.404)
Age					0.031
					(0.052)
FriendsNotAtSchool					0.013
					(0.052)
MoreThan1handicap (0 = No, 1 = Yes)					-0.009
					(0.429)
N	138	103	103	103	103

*Standard errors in parentheses*.

** p<0.*05*

* p<0.*1*

In conclusion, these regression results support the idea that attending regular school did not negatively affect the social inclusion of the SEN students assessed by the IOS measure. The dummy variable Regular was not significant, while the SchoolInclusion variable was highly significant in all models: a school scoring higher on the inclusive characteristics was linked to higher social inclusion as measured by the IOS. At the same time, the interaction effect of the SchoolInclusion variable with the school type remained insignificant. Therefore, we conclude that the school type did not affect the SEN student’s social inclusion, only the inclusive culture of the school itself, independent of the school type.

## Discussion

Our study presents a methodological innovation for measuring social inclusion by a simple pictorial measure IOS Scale, using the perception of being socially included as a relevant input to evaluate the success of failure of social inclusion of SEN students at school. This measure has been extensively validated in previous research, and has an excellent track record of addressing both close social relationships and the feeling of belonging of an individual toward a specified “other” category, be it another group of people, a cultural category, or even oneself in another point in time [59–62]. The feeling of belonging is central for the definition of social inclusion, and we suggest that this scale is a suitable measure of social inclusion in various contexts, e.g. at home or school. Our observation that the IOS Scale correlates with a loneliness measure obtained for the same context (school), but not with a loneliness measured in a different context (home), supports that the IOS Scale taps into the social inclusion aspect of belonging, and not merely into the feeling of being alone.

Using the IOS Scale as a measure of social inclusion represents a shift in focus from purely relying on sociometric methods for assessing social inclusion, such as peer acceptance, interactions with others, peer ranking, and bilateral friendships [6, 63–65]. Individuals obtaining lower values for such measures are identified as being less socially included. We advocate complementing such measures with subjective measures like the IOS measure, to incorporate the voice and perceptions of the involved individuals, or their parents/care-takers as in our study, with the goal of furthering the discussions on success or failure of inclusion in education.

Our study has some limitations that need to be addressed and which demand more research. Most importantly, we obtained data by using parental proxy reports. There is a long line of research in health and assessment of quality of life literature, suggesting that parental proxy reports do not always achieve fit with child reports [66]. Parental characteristics, or family context [67], may affect the extent to which a parent is able to empathize with a child. At the same time, research on subjective well-being of children with disabilities shows that an overlap of child and parent reports may be rather satisfactory in the field of perceived friendship [52], though direct reports are preferable whenever possible. In this study, we have benefited from collaboration with organizations collecting information from parents, which resulted in a convenience sample of parents reporting on their children. This allowed us to take a first step in using the IOS Scale to learn how the social inclusion of SEN students is perceived by their parents. However, this approach should not be understood as a perfect substitute for reports of those involved in the education contexts of various types, the SEN students themselves. Focus on data collection from the SEN student population, along with self-reports, is a desirable route for future research to give voice to the SEN students themselves, as is research that further clarifies the fit between parental and child reports of the IOS measure.

Additionally, this study’s recruitment channel, via organizations supporting children’s rights, might have resulted in the overrepresentation of parents actively seeking education contexts fitting their children, and follow-up studies should focus on parents underrepresented in these groups. Two related concerns are (i) a selection effect, which could be present if parents of SEN students more likely to achieve social inclusion are also more likely to apply for admission into regular schools, and (ii) a projection effect, arising if parents across different school types adjust differently to the perceptions of their children’s social inclusion. Such self-confirming bias, for example, could prevent parents of children admitted into regular schools from recognizing the failing social inclusion of their children in the selected educational setting. We use the variables FriendsNotAtSchool and ParentalInclusion to address these issues in our regression analysis, and the regression results suggest that these effects do not drive our results.

The main result of our study is that social inclusion of SEN students, as measured by the parental reports of the IOS measure, was not significantly predicted by the school category, which can be interpreted as no negative impact of the inclusion of SEN students in regular schools. At the same time, the variable SchoolInclusion, based on the inclusive characteristics was a significant predictor of a higher perceived social inclusion of SEN students. This variable is composed of answers to questions on how welcoming the school was, supporting collaboration and inclusion of all learners, making decisions to protect each child’s well-being, and making didactic choices that acknowledge diversity and support collaboration. In our sample, the regular schools scored higher on this variable than the special schools; however, the fact that the dummy variable categorizing the schools remained insignificant suggests that there is a variation among the schools within this category, so that the SchoolInclusion variable explains social inclusion. This leads us to point out that promoting school characteristics aligned with inclusive education is a promising pathway to SEN students’ social inclusion.

## Conclusions

Researchers have repeatedly raised concerns that letting SEN students attend regular schools might fail to achieve their social inclusion [5, 64, 65, 68–71]. These studies could be taken as a warning sign not to overstate the value of inclusive education for achieving the goal of social inclusion. We assert that such warnings about SEN students’ social inclusion do not address whether the special school environments are more effective in realizing the social inclusion of SEN students. This can be answered by studying social inclusion of SEN students across school contexts. To address this question, we introduce a new tool for the methodological toolbox of social inclusion research.

We propose to adopt a simple pictorial IOS Scale [17] to assess social inclusion as the subjective perception of being included. In this, we are inspired by the literature on subjective well-being and its contribution policy design, and by the human rights perspective on giving voice to the individuals affected by the policies, such as education policies [72]. We hope that the introduction of subjective social inclusion measures can further help to understand the impact of policies promoting social inclusion, and constructively contribute to social inclusion research.

In order to contribute to this objective, we applied the IOS Scale to compare the social inclusion of SEN students across school types. We observed that parents of SEN students attending regular schools did not perceive their social inclusion to be lower compared to reports of parents of SEN students attending special schools. Parental reports also indicated that SEN students scored low on inclusion at both types of schools: this identifies an undesirable phenomenon that deserves attention. We also observed that SEN students were generally perceived by their parents as less lonely at school than at home, independent of the school type. School also played an important role as a friendship incubator for SEN students. SEN students in our sample formed most friendships at school, independent of the school type. However, SEN students attending regular schools had more friends, and their friends lived closer to them than the friends of SEN students attending special schools. This indicates that letting SEN students attend regular schools had an important positive spillover impact on their social inclusion, by forming their social networks locally, in the communities where they live.

We consider the relevance of these findings to lie in the realm of policy development, since they offer new insights into the social inclusion of SEN students by comparing their social inclusion across school types. In the present comparison, letting SEN students attend regular schools was not associated with negative consequences for their social inclusion. On that note, our findings highlight the importance of interventions aiming at schools’ inclusive practices, as those were found to increase social inclusion.

## Supporting information

S1 File (DOCX)

S2 File (DOCX)

S3 File (SAV)
