# Supplementary material for: Social inclusion of students with special educational needs assessed by the Inclusion of Other in the Self scale
Source: PLoS One. 2021 Apr 28;16(4):e0250070. doi: 10.1371/journal.pone.0250070 (PMC8081169; doi:10.1371/journal.pone.0250070)
Supplement: S1 File — (DOCX) [file pone.0250070.s001.docx]

**Vragenlijst**

Ik ben:
Ouder of verzorger van een kind met een beperking of chronische ziekte tussen de 4 en 20 jaar
Ja/Nee

*Heeft u meerdere kinderen met een beperking of chronische ziekte, dan vragen wij u voor ieder kind apart een vragenlijst in te vullen. Nadat u de vragenlijst volledig heeft ingevuld voor één kind, vult u dezelfde vragenlijst opnieuw in voor uw andere kind(eren).*

Voordat u de vragen invult, vragen wij eerst onderstaande goed te lezen.

*Ik verklaar hierbij op een voor mij duidelijke wijze te zijn ingelicht over het onderzoek, zoals beschreven in de uitnodigingsmail voor dit onderzoek. Ik verklaar mij akkoord met het verzamelen van gegevens, door middel van een vragenlijst, wat door Ieder(in) zal worden uitgevoerd. Er vindt een verwerking van deze gegevens plaats door Ieder(in), Patiënten Federatie Nederland en Landelijk Platform GGz. Zij dragen zorg voor het veilig beheren van deze gegevens.*

***√ ik begrijp de bovenstaande tekst en ga akkoord met deelname aan het onderzoek van Ieder(in)***

*Uw gegevens zijn ook interessant voor extra wetenschappelijk onderzoek dat de Radboud Universiteit wil doen. Ook daarvoor vragen we uw toestemming. Daarover gaat de volgende tekst.*

*Ik verklaar hierbij dat ik geen bezwaar heb wanneer Ieder(in) mijn geanonimiseerde gegevens, dus zonder persoonsgegevens, deelt met de Radboud Universiteit Nijmegen voor wetenschappelijk onderzoek.*

*Ik stem geheel vrijwillig in met deelname aan dit wetenschappelijk onderzoek. Ik behoud daarbij het recht deze toestemming weer in te trekken zonder dat ik daarvoor een reden hoef op te geven. Ik besef dat ik op elk moment mag stoppen met deelname aan het onderzoek.*

*Als mijn onderzoeksresultaten worden gebruikt in wetenschappelijke publicaties, of op een andere manier openbaar worden gemaakt, dan zal dit volledig geanonimiseerd gebeuren. Mijn persoonsgegevens worden niet gedeeld met de Radboud Universiteit of door derden ingezien zonder mijn uitdrukkelijke toestemming.*

*Als ik meer informatie wil, nu of in de toekomst, dan kan ik contact opnemen met Ieder(in) via g.boezaard@iederin.nl of bellen 06-461 323 51.*

***√ ik begrijp de bovenstaande tekst en ga akkoord met deelname aan het onderzoek van de Radboud Universiteit***

Q1: Welke beperking of chronische ziekte heeft uw kind? (meerdere keuzes mogelijk)

- visuele beperking
- auditieve beperking
- lichamelijke beperking
- verstandelijke beperking
- psychische beperking
- chronische ziekte, namelijk: .........
- anders, namelijk:.........

Q2: Welke vorm van onderwijs volgt uw kind op dit moment?

- regulier basisonderwijs
- speciaal (basis) onderwijs
- regulier voortgezet onderwijs
- voortgezet speciaal onderwijs
- praktijkonderwijs
- anders, namelijk: ......

Q3: Welke van de volgende plaatjes beschrijft het beste de huidige relatie van uw kind met de andere kinderen op zijn of haar school?

Anderen

Mijn kind

Anderen

Mijn kind

Anderen

Mijn kind

Anderen

Mijn kind

Mijn kind

Anderen

Mijn kind

Anderen

Anderen

Mijn kind

Ik vind de vraag te lastig. Ik sla deze over.

Q4: Geef aan wat op uw kind van toepassing is. (Helemaal eens – eens – neutraal – oneens – helemaal oneens – weet ik niet)

- Mijn kind voelt zich gewaardeerd door andere kinderen op school
- Mijn kind voelt zich alleen op school
- Mijn kind krijgt hulp van andere kinderen op school
- Mijn kind voelt zich buitengesloten op school
- Mijn kind is graag met anderen in de pauzes
- Mijn kind heeft weinig tot géén ruzie met kinderen op school
- Mijn kind is eenzaam op school

Q5: Geef aan wat op uw kind van toepassing is. (Helemaal eens – eens – neutraal – oneens – helemaal oneens – weet ik niet)

- Mijn kind voelt zich thuis gewaardeerd door vrienden en familie
- Mijn kind voelt zich thuis alleen
- Mijn kind krijgt thuis hulp van vrienden en familie
- Mijn kind voelt zich thuis buitengesloten
- Mijn kind is thuis graag met anderen
- Mijn kind heeft thuis weinig tot géén ruzie
- Mijn kind is thuis eenzaam

Q6: Onderstaande vraag gaat over wat u weinig of veel vrienden zou vinden. Vul bij iédere zin een cijfer in:

- Hoeveel vrienden zou uw kind moeten hebben zodat u zou zeggen:
  mijn kind heeft heel weinig vrienden:
  minder dan ___ vriend/en
- Hoeveel vrienden zou uw kind moeten hebben zodat u zou zeggen:
  mijn kind heeft weinig vrienden:
  ten minste____ vrienden
- Hoeveel vrienden zou uw kind moeten hebben zodat u zou zeggen:
  mijn kind heeft een normaal aantal vrienden:
  ten minste____ vrienden
- Hoeveel vrienden zou uw kind moeten hebben zodat u zou zeggen:
  mijn kind heeft redelijk veel vrienden:
  ten minste____ vrienden
- Hoeveel vrienden zou uw kind moeten hebben zodat u zou zeggen:
  mijn kind heeft heel veel vrienden:
  ________of meer vrienden
- Ik vind deze vraag moeilijk te beantwoorden, ik sla deze over

Q7: De volgende vragen gaan over de school waar uw kind naar toe gaat. Kies bij elke stelling één van de vijf mogelijke antwoorden (helemaal eens, eens, neutraal, oneens, helemaal oneens, of weet ik niet).

- Mijn kind gaat graag naar deze school
- Mijn kind leert veel op deze school
- Iedereen is welkom op deze school
- Kinderen worden betrokken bij hun eigen leerweg
- De school helpt de leerlingen het beste uit zichzelf te halen
- De school werkt op een gelijkwaardige manier met mij als ouder/verzorger samen
- Op deze school is extra ondersteuning goed geregeld
- Op school is er aandacht voor dat kinderen elkaar niet pesten
- Alle kinderen worden gestimuleerd om deel te nemen aan wat er in de klas gebeurt
- Kinderen leren over diversiteit op deze school
- Kinderen leren op school door met elkaar samen te werken
- Alle kinderen op deze school nemen deel aan buitenschoolse activiteiten (kamp, schoolreisjes, enz.)

Q8: Hoeveel vrienden heeft uw kind op school?

- Geen vriend
- 1 vriend
- meer dan 1 vriend, namelijk:_____
- weet ik niet

Q9: Hoeveel vrienden heeft uw kind die niet op dezelfde school met hem/haar zitten?

- Geen
- 1
- meer dan 1, namelijk:_____
- weet ik niet

Q10: Geef antwoord op de volgende vragen:

- Alle vrienden van mijn kind hebben dezelfde beperking/chronische aandoening als hij/zij. JA / NEE
- Sommige vrienden van mijn kind hebben een andere beperking/chronische aandoening dan hij/zij. JA / NEE
- Sommige vrienden van mijn kind hebben geen beperking/chronische aandoening. JA / NEE
- De meeste vrienden van mijn kind zijn: jonger dan mijn kind / net zo oud als mijn kind / ouder dan mijn kind.
- De meeste vrienden van mijn kind wonen: in dezelfde buurt / in dezelfde woonplaats / in een andere woonplaats

Q11: Hoe vaak wordt uw kind door een andere leerling uitgenodigd voor een verjaardagsfeestje?

• Nooit

• 1 keer per schooljaar

• meer dat 1 keer, namelijk:____

• anders, namelijk:______

Q12: Nodigt uw kind wel eens andere leerlingen uit voor zijn/haar eigen verjaardag?

• Nooit

• 1 keer per schooljaar

• meer dat 1 keer, namelijk:____

• anders, namelijk:______

Q13: Wat is voor uw kind belangrijk om erbij te horen op school?

Q14: Wilt u ons nog iets vertellen over de rol die de school van uw kind speelt in zijn/haar sociale leven?

Q15: Hoe oud is uw kind waarvoor u deze vragenlijst invult? _________

Q16: Mijn kind is een

- meisje
- jongen

Q17: Woont uw kind bij u thuis?

- ja
- nee, namelijk:_________

Q18: Hoe is het gezin van uw kind samengesteld?

- mijn kind is enig kind
- mijn kind heeft nog ____ broers en ---- zussen
- en is de jongste
- en is de oudste
- anders

Q19: Bent u lid van een vereniging, stichting, organisatie gerelateerd aan de beperking of chronische aandoening van uw kind:

- ja, namelijk (u kunt meerdere organisaties invullen): ______
- nee

Wil je op de hoogte gehouden worden over de uitkomsten van dit onderzoek?

- ja
- nee

Wil je uitgenodigd worden voor een vervolgbijeenkomst, die we in aanvulling op het onderzoek gaan organiseren?

- ja
- nee

*Heb je op een van de laatste drie vragen ‘ja’ geantwoord, vul dan hier je gegevens in:*

- Naam
- Telefoonnummer:
- E-mailadres:
