# Supplementary material for: Social inclusion of students with special educational needs assessed by the Inclusion of Other in the Self scale
Source: PLoS One. 2021 Apr 28;16(4):e0250070. doi: 10.1371/journal.pone.0250070 (PMC8081169; doi:10.1371/journal.pone.0250070)
Supplement: S2 File — (DOCX) [file pone.0250070.s002.docx]

**Online distributed questionnaire on social inclusion of special education needs students assessed by the Inclusion of the Self in Other**

Please answer:

I am a parent or care-taker of a child with handicap in the age between 4 and 20.

Yes (questionnaire continues) / No (questionnaire stops)

If yes, then:

*If you are a parent of more than one child with handicap or chronic condition, then please fill in a separate questionnaire for each of them. You can start a new questionnaire after you have finished one*.

Before you start the questionnaire, please read carefully the following text:

*I declare that I received information about this research, described in the invitation email, in an understandable way. I agree with collecting the information that I provide in this questionnaire. The information I provide will be processed by (INSERT). They are also responsible for safe storage of the information I provide.*

***√ I understand the above written text and give my consent to the participation in the research of (INSERT).***

The information you will provide is also relevant for scientific research realized by [INSERT]. We also ask if you want to give permission for this. Please read the following text about that.

*I herewith give consent that Ieder(In) shares the information that I provided, in anonymized form, that is, without my personal information, with the [INSERT], for the purpose of scientific research. I give my consent to participate in this scientific research. I retain the right to withdraw my consent at any time, without giving the reason. I am aware that I can stop the participation in the research at any moment.*

*If my data is used for scientific publication, or in any other public way, it will be in a fully anonymized form. My personal information will not be shared with the [INSERT], or by third parties, unless I give explicit consent.*

*If you want to receive more information, now or in the future, you can contact [INSERT].*

***√ I understand the above written text and give my consent to the participation in the research of [INSERT].***

Q1: What handicap does your child have? (more choices possible)

- visual handicap
- auditive handicap
- physical handicap
- cognitive handicap
- psychic handicap
- chronic disease, in particular: .........
- other, in particular:.........

Q2: What type of school does your child attend currently?

- mainstream elementary school
- mainstream secondary school
- special (elementary) school
- special secondary school
- vocational school
- other, in particular:.........

Q3: Which one of the following figures does describe the current relation of your child with other schoolmates at his/her school? Choose one of the figures below, or select: “I find this question too difficult, I do not provide an answer.”

Other children

My child

Other children

My child

Other children

My child

Other children

My child

My child

Other children

My child

Other children

Other children

My child

I find this question too difficult, I do not provide an answer.

Q4: Answer the questions about your child: (Fully agree, Agree, Neutral, Disagree, Fully disagree, I do not know)

- My child feels to be appreciated by other schoolmates at school.
- My child feels alone at school.
- My child gets help from other schoolmates at school.
- My child feels left out of things at school.
- My child likes to be with other schoolmates during the breaks at school.
- My child has little up to none conflict with my schoolmates at school.
- My child is lonely at school.

Q5: Answer the following questions about situation at home. (Fully agree, Agree, Neutral, Disagree, Fully disagree, I do not know)

- My child is well-liked by friends and family.
- My child is lonely at home.
- My child gets help from family and friends.
- My child feels left out of things at home.
- My child likes to be with family members.
- My child gets along well with family members.
- My child feels alone at home.

Q6: The next question is about what you perceive as having few or many friends. Fill in one number in each line.

- How many friends should your child have so that you would say;

*my child has very few friends*: less than ___ friends

- How many friends would your child have so that you would say:

*my child has few friends:* at least ____ friends

- How many friends would your child have so that you would say:

*my child has normal number of friends:* at least ____ friends

- How many friends would your child have so that you would say:

*my child has quite large number of friends:* at least ____ friends

- How many friends would your child have so that you would say:

*my child has very large number of friends: ________*of more friends

- I find this question too difficult to answer, I skip it.

Q7: The following questions are about the school that your child attends. Choose for each statement one of the five possible answers (Fully agree, Agree, Neutral, Disagree, Fully disagree); or I do not know.

- My child enjoys being in this school.
- My child learns a lot in this school.
- Everyone is made feel welcome at this school.
- Children are actively involved in their own learning.
- My school helps all students to do their best.
- The school works in partnership with me as parent/care-taker.
- All forms of support are co-ordinated at this school.
- Everything possible is done to stop bullying among children in the school.
- Lessons encourage the participation of all children at the school.
- Lessons at this school develop an understanding of difference.
- Children learn collaboratively at this school.
- All children take part in activities outside the classroom (camp, school trips etc.)

Q8: How many friends does your child have at school?

- None
- One
- More than one, namely:_____
- I do not know.

Q9: How many friends does your child have that do not attend the same school?

- None
- One
- More than one, namely:_____
- I do not know.

Q10: Please answer:

- All friends of my child have the same handicap/chronic disease as s/he: Yes / No
- Some friends of my child have the another handicap/chronic disease than s/he: Yes / No
- Some friends of my child do not have handicap/chronic disease: Yes / No
- Most friends of my child are: Younger than my child / same age as my child / older than my child
- Most friends of my child live: In the same neighbourhood as my child/ In the same place (city, village) as my child / In another place(city, village) than my child

Q11: How often is your child invited to a birthday celebration by a schoolmate from his/her school?

- Never
- Once in a school year
- More than once in a school year, namely:____
- Other answer:______

Q12: Does your child invite other schoolmates from his/her school for own birthday celebration?

- Never
- Once in a school year
- More than once in a school year, namely:____
- Other answer:______

Q13: What does your child need to have a feeling of belonging at school? (open question)

Q14: Would you share with us what is the role of school in the social life of your child? (open question)

Q15: How old is the child that you filled in this questionnaire about?

Q16: My child is: a girl / a boy

Q17: Does your child live with you at home? Yes/ No, in particular:___

Q18: What is the composition of your family?

- My child is the only child
- My child has …. brothers and … sisters
- And is the youngest
- And is the oldest
- Other answer

Q19: Are you a member of an organization or foundation related to the handicap/ chronic disease of your child: Yes: please give names (multiple answers possible)/ No

Q20: Do you want receive information about the outcomes of this research? Yes/No

Q21: May we contact you with additional questions, or ask for an interview? Yes/No

Q22: Do you want to be invited for a follow-up meeting that we will organize additionally to the questionnaire research? Yes/No

*Q23: If you answered “yes” at one of the previous three questions, then please fill in your information here:*

- Name
- Phone number
- Email address:
